# Prognostic Value of Quantitative Stress Perfusion Cardiac Magnetic Resonance

**DOI:** 10.1016/j.jcmg.2017.07.022

**Published:** 2018-05

**Authors:** Eva C. Sammut, Adriana D.M. Villa, Gabriella Di Giovine, Luke Dancy, Filippo Bosio, Thomas Gibbs, Swarna Jeyabraba, Susanne Schwenke, Steven E. Williams, Michael Marber, Khaled Alfakih, Tevfik F. Ismail, Reza Razavi, Amedeo Chiribiri

**Affiliations:** a School of Biomedical Engineering and Imaging Sciences, King’s College London, London, United Kingdom; b Bristol Heart Institute, Bristol, United Kingdom; c Department of Cardiology, King’s College Hospital, London, United Kingdom; d SCOSSiS Statistical Consulting, Berlin, Germany; e Cardiovascular Division, King’s College London, London, United Kingdom

**Keywords:** cardiac magnetic resonance, perfusion, prognosis, quantitative perfusion analysis, AHA, American Heart Association, CABG, coronary artery bypass grafting, CMR, cardiac magnetic resonance, IQR, interquartile range, LGE, late gadolinium enhancement, MBF, myocardial blood flow, MPR, myocardial perfusion reserve, PCI, percutaneous coronary intervention, PET, positron emission computed tomography, ROI, region of interest, SCD, sudden cardiac death, SPECT, single-photon emission computed tomography

## Abstract

**Objectives:**

This study sought to evaluate the prognostic usefulness of visual and quantitative perfusion cardiac magnetic resonance (CMR) ischemic burden in an unselected group of patients and to assess the validity of consensus-based ischemic burden thresholds extrapolated from nuclear studies.

**Background:**

There are limited data on the prognostic value of assessing myocardial ischemic burden by CMR, and there are none using quantitative perfusion analysis.

**Methods:**

Patients with suspected coronary artery disease referred for adenosine-stress perfusion CMR were included (n = 395; 70% male; age 58 ± 13 years). The primary endpoint was a composite of cardiovascular death, nonfatal myocardial infarction, aborted sudden death, and revascularization after 90 days. Perfusion scans were assessed visually and with quantitative analysis. Cross-validated Cox regression analysis and net reclassification improvement were used to assess the incremental prognostic value of visual or quantitative perfusion analysis over a baseline clinical model, initially as continuous covariates, then using accepted thresholds of ≥2 segments or ≥10% myocardium.

**Results:**

After a median 460 days (interquartile range: 190 to 869 days) follow-up, 52 patients reached the primary endpoint. At 2 years, the addition of ischemic burden was found to increase prognostic value over a baseline model of age, sex, and late gadolinium enhancement (baseline model area under the curve [AUC]: 0.75; visual AUC: 0.84; quantitative AUC: 0.85). Dichotomized quantitative ischemic burden performed better than visual assessment (net reclassification improvement 0.043 vs. 0.003 against baseline model).

**Conclusions:**

This study was the first to address the prognostic benefit of quantitative analysis of perfusion CMR and to support the use of consensus-based ischemic burden thresholds by perfusion CMR for prognostic evaluation of patients with suspected coronary artery disease. Quantitative analysis provided incremental prognostic value to visual assessment and established risk factors, potentially representing an important step forward in the translation of quantitative CMR perfusion analysis to the clinical setting.

## Graphical abstract

**Figure:**
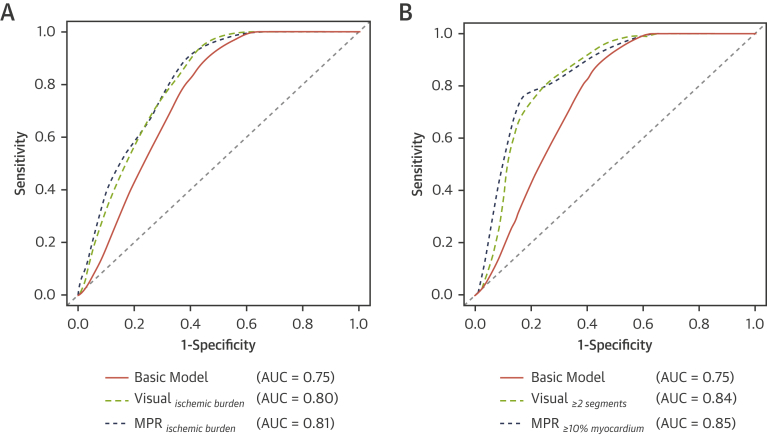

In recent years, stress perfusion cardiac magnetic resonance (CMR) has become one of the methods of choice for the diagnosis of coronary artery disease based on high diagnostic accuracy, lack of ionizing radiation, and the ability to simultaneously assess cardiac function, myocardial perfusion, and viability 1, 2, 3. In the clinical setting, perfusion CMR is assessed qualitatively with visual analysis (4). There are data that show that ischemia detection by visual assessment performs similarly or is at least noninferior to other noninvasive modalities such as positron emission tomography (PET) or single-photon emission computed tomography (SPECT) 5, 6, 7.

There is a growing body of evidence that shows that the mere presence of ischemia is of prognostic value 8, 9, 10, 11, 12; however, in recent years, the burden of ischemia has become an important focus 13, 14. SPECT allows quantification of relative ischemic burden in terms of percentage of ischemic myocardium, and accepted thresholds have been shown to be of prognostic value in large nuclear data sets 13, 15. The thresholds of ischemic burden used in SPECT have been extrapolated to CMR 16, 17; however, evidence regarding the validity of these thresholds for CMR is lacking.

CMR perfusion quantification has been shown to be feasible and has been validated against invasive and noninvasive modalities, including fractional flow reserve (18) and PET (19). Although both visual and quantitative analysis can provide data on ischemic burden, there are data that suggest the diagnostic superiority of quantitative CMR perfusion analysis, specifically in the setting of multivessel coronary disease (20). A direct comparison between visual and quantitative perfusion CMR in terms of prognostic benefit has yet to be performed.

The main aim of this study was to assess the prognostic value of quantitative CMR perfusion analysis. Furthermore, we aimed to investigate the validity of accepted consensus-based thresholds for ischemic burden in the setting of adenosine stress perfusion CMR.

## Methods

### Study design

Patients with suspected coronary artery disease referred on a clinical basis to King’s College London CMR Service at St Thomas’ Hospital (Guy’s and St Thomas’ NHS Trust) between January 2009 and January 2015 were considered for enrollment. Consecutive patients who underwent a clinical CMR scan that included assessment of cardiac function, ischemia testing with high-resolution dual-bolus adenosine stress, and resting perfusion imaging, followed by late gadolinium enhancement (LGE) imaging were included. Patients in whom regadenoson was used were excluded because the long half-life of the drug has the potential to result in the acquisition of resting perfusion images with a degree of residual hyperemia, which results in the underestimation of myocardial perfusion reserve (MPR) 21, 22.

The study was conducted in accordance with the Declaration of Helsinki (2000) and was approved by the National Research Ethics Service. All patients provided written informed consent.

### Study endpoint and patient follow-up

The start of follow-up was defined as the date of the CMR scan. The primary endpoint was a composite of cardiovascular death (death due to myocardial infarction, heart failure, or ventricular arrhythmia), nonfatal myocardial infarction (acute coronary syndrome with associated electrocardiographic changes and elevation of serum biomarkers of myocardial necrosis to >99th percentile of the assay upper limit of normal [23]), aborted sudden cardiac death (SCD) (documented ventricular fibrillation or sustained ventricular tachycardia with hemodynamic compromise requiring defibrillation or cardioversion not associated with acute myocardial infarction), and late revascularization (percutaneous coronary intervention [PCI] or coronary artery bypass grafting [CABG] >90 days after the start of follow-up). To avoid the potential effect of a positive CMR result leading to early revascularization, we excluded procedures within 90 days of the CMR scan from the definition of the endpoint revascularization (24). Patients who did not experience events were censored at the point of last follow-up. When patients experienced >1 event, only the first event was considered.

Follow-up was performed through interrogation of electronic patient records and patient notes. All events were adjudicated by consensus of 3 physicians blinded to CMR data. Mortality and cause of death were obtained from the Office for National Statistics and through review of medical records, death certificates, and post-mortem data where available.

### Image acquisition

Patients were asked to refrain from nicotine and caffeine-containing foods and beverages for 24 h before the scan. Standard 2-, 3-, and 4-chamber cine images were acquired during breath hold. Contiguous short-axis slices were acquired from the base to the apex for calculation of left ventricular volumes, function, and mass. Following 4 min of adenosine (140 μg/kg/min, increased to 210 μg/kg/min if there was an inadequate response, which was considered an increase in heart rate at peak stress of <10% above baseline [25]), stress perfusion data were acquired in 3 short-axis slices with a saturation-recovery *k*-*t* sensitivity encoding accelerated gradient-echo method (26), which covered 16 of the standard myocardial segments (segment 17 was excluded).

A dual-bolus contrast agent scheme was used to correct for signal saturation of the arterial input function as previously described (27). In brief, 0.0075 mmol/kg gadobutrol (Gadovist, Bayer, Berlin, Germany) was administered as a pre-bolus at peak stress with imaging of the arterial input function. First-pass perfusion data were then acquired after the injection of 0.075 mmol/kg gadobutrol at 4 ml/s, followed by a 20-ml saline. Resting perfusion imaging was performed after a minimum of 15 min following the stress acquisition before acquisition of LGE imaging according to Society for Cardiovascular Magnetic Resonance (SCMR) guidelines (28). LGE imaging was performed after a further dose of contrast agent, to a total dose of 0.2 mmol/kg.

### Image analysis

Ventricular volumes, atrial size, and left ventricular mass were calculated and normalized to body surface area (CVI42, v5.1.1, Circle Cardiovascular Imaging, Calgary, Ontario, Canada). Segmental mass was measured in end-diastole and expressed as percentage of total myocardial mass.

Visual assessment was performed using stress and resting perfusion, and LGE images by the consensus of 2 SCMR level III accredited physicians using the standard 16-segment American Heart Association (AHA) model and standardized reporting criteria. Scans were reported visually on a clinical basis; therefore, baseline clinical data were available to the observers who performed visual assessment.

A perfusion abnormality was defined visually according to the criteria previously described in the MR-INFORM (MR Perfusion Imaging to Guide Management of Patients With Stable Coronary Artery Disease) trial (17), as a delayed wash-in of contrast persisting for ≥5 dynamics in ≥1 AHA segments compared with normal remote myocardium. Each AHA segment was subdivided into an endocardial and epicardial half, which resulted in a total of 32 segments to allow more accurate calculation of ischemic burden. Segments in which LGE was present were excluded from ischemia assessment.

Surface coil intensity correction was performed before quantification using pre-contrast imaging data (29). Time signal-intensity curves were extracted using commercially available software (CVI42). Quantitative analysis was performed blinded to baseline clinical data.

Quantitative perfusion analysis was performed by Fermi-constrained deconvolution according to the previously described methods 30, 31, in which time-signal intensity curves for the tissue impulse response function, *h*(*t*), were fitted to the Fermi function using a Marquardt-Levenberg nonlinear least-squares algorithm according to the following analytical expression:

$$h\left(t\right)=R\left[\frac{1}{e^{\left(t-\tau_{0}-\tau_{d}\right)k}+1}\right]u\left(t-\tau_{d}\right)$$

by letting *k*, *R*, and *τ*_0_ vary and keeping *τ*_*d*_ fixed. In the preceding equation, *u*(*t* − *τ*_*d*_) is the unit step function. The *τ*_*d*_ accounts for the delay time between the appearance of the signal in the left ventricular blood pool and myocardial region of interest (ROI); *τ*_0_ characterizes the width of the shoulder of the Fermi function during which little or no contrast agent had left the ROI. *R* is the index of contrast agent influx parameter, and *k* represents the decay rate of *h*(*t*) due to contrast agent washout. Using the preceding equation, myocardial blood flow (MBF) estimates are calculated as *h*(*t*) at *t* = 0. MPR was calculated as the ratio between stress and resting MBF estimates (32). Ischemia was defined as segments with MPR <1.5, according to previously validated criteria (18).

### Statistical methods

Statistical analysis was performed by 2 of the authors (S.S. and T.I.). Data for categorical variables are presented as frequencies and percentages. Data for continuous variables are presented as mean ± SD or as median and interquartile range (IQR) depending on normality. The study cohort was stratified according to the presence or absence of events during follow-up.

Differences between categorical variables were evaluated using Fisher exact test. Comparisons between continuous variables were performed with the independent samples Student’s *t*-test or using the Wilcoxon rank-sum test as appropriate. Two-tailed p values <0.05 were considered significant.

Survival risk classifiers were built using penalized Cox proportional hazards models with a L1-(Lasso) penalty. Double-loop, cross-validation with 500 restarts was used to obtain robust unbiased estimates of predictive strength as outlined by Simon et al. (33). A baseline model was built containing established prognostic variables (age, sex, and LGE) as unpenalized independent variables. Left ventricular ejection fraction, revascularization within 90 days, and pre-scan revascularization (34) also entered the baseline model and were subject to penalization. None of these penalized variables were found to provide predictive information, and therefore, were not retained in the final model specification. The baseline model was then extended to include visual ischemic burden or MPR ischemic burden. The predictive performance of the resulting enriched models was evaluated by 10-fold cross-validation with 500 restarts (33). Survival probabilities were derived for each patient for the 2-year time point. The 2-year time point was chosen on the basis of ≥75% of the cohort having a follow-up of 869 days (2.4 years). Cross-validated, receiver-operating characteristic curves were created for each model at 2 years, and the associated cross-validated area under the curve was determined (35). Patients were classified into 3 categories: low (<1%), intermediate (1% to 3%), and high (>3%) risk as specified by current American College of Cardiology guidelines (3). Cross-validated categorical net reclassification improvement and integrated discrimination improvement reclassification metrics were then computed for the 2 perfusion models in relation to the baseline model (36).

After establishing the independent predictive value of each measure of perfusion as a continuous covariate, the perfusion measures were dichotomized using a threshold of ≥2 segments for visual ischemia and ≥10% for ischemic MPR (16). Model performance was reassessed as previously described using cross-validated Cox regression and reclassification metrics for the 2-year time point. Kaplan-Meier-curves were produced for time to the composite endpoint to visualize the discriminative power of LGE and perfusion measurements. All statistical analysis was performed with R version 3.3.0 (R Core Team, R Foundation for Statistical Computing, Vienna, Austria).

## Results

A total of 434 patients met the inclusion criteria: 24 (5.5%) were lost to follow-up, and 15 (3.5%) were excluded due to poor image quality. The baseline clinical and CMR characteristics of the final cohort of 395 patients stratified by event status are listed in Table 1. Patients tended to be middle-aged (58 ± 13 years) and mostly men (70%); most had hypertension (59.5%) or hypercholesterolemia (54.7%), and approximately 1 in 5 had diabetes (19.7%). Approximately one-third of the patients had had previous revascularization either by PCI or CABG. Approximately one-third (34.9%) of the patients had stress-induced perfusion abnormalities on visual assessment, and 35.4% were positive for LGE. In total, 40 patients underwent revascularization within 90 days (n = 7 CABG, n = 33 PCI, at a median of 30 and 36 days, respectively, post-CMR), and these events were excluded from subsequent analysis.

**Table 1:** Baseline Clinical and CMR Characteristics

	No Event (n = 343; 86.8%)	Event (n = 52; 13.2%)	Total (N = 395; 100%)	p Value
Age, yrs	57.6 ± 13.2	63.3 ± 11.8	58.3 ± 13.1	0.019
Male	226 (65.9)	51 (98.1)	277 (70.1)	<0.001
Body mass index, kg/m^2^	1.95 ± 0.23	2.00 ± 0.20	1.96 ± 0.23	0.612
Risk factors				
Diabetes mellitus	66 (19.2)	12 (23.5)	78 (19.7)	0.575
Hypertension	201 (58.6)	34 (66.7)	235 (59.5)	0.368
Current smoker	63 (18.4)	10 (19.2)	73 (18.5)	0.849
Ex-smoker	31 (9.0)	4 (7.7)	35 (8.9)	1.000
Hypercholesterolemia	180 (52.5)	36 (72)	216 (54.7)	0.025
Previous PCI/CABG	101 (29.2)	34 (65.4)	135 (34.2)	<0.001
PCI/CABG within 90 days	36 (10.5)	4 (7.7)	40 (10.1)	0.804
Atrial fibrillation	36 (10.5)	10 (19.2)	46 (11.6)	0.100
CMR characteristics				
LVEDV, ml	152 (130–183)	156 (125–195)	153 (129–183)	0.462
Indexed LVEDV, ml/m^2^	78 (67–91)	79 (65–96)	79 (67–91)	0.790
LVESV, ml	60 (47–82)	65 (41–97)	60 (46–84)	0.223
Indexed LVESV, ml/m^2^	30 (24–40)	32 (22–47)	31 (24–41)	0.374
LVEF, %	60 (53–66)	59 (44–67)	60 (52–66)	0.121
LV mass, g	107 (88–134)	115 (97–134)	109 (90–134)	0.086
Indexed LV mass, g/m^2^	55 (46–66)	60 (50–67)	56 (47–56)	0.075
RVEDV, ml	149 (126–174)	143 (114–170)	147 (124–174)	0.185
Indexed RVEDV, ml/m^2^	75 (64–87)	73 (60–88)	31 (23–38)	0.178
RVESV, ml	62 (44–77)	57 (46–78)	61 (44–77)	0.671
Indexed RVESV, ml/m^2^	31 (23–39)	31 (22–38)	31 (23–38)	0.610
LA size, cm^2^	23 (20–26)	22 (18–27)	23 (19–26)	0.944
RA size, cm^2^	20 (17–23)	21 (18–24)	20 (17–23)	0.464
Resting heart rate, beats/min	71.8 ± 14.2	68.7 ± 13.5	70.8 ± 14.0	0.182
Stress heart rate, beats/min	96.8 ± 17.4	90.9 ± 16.1	95.6 ± 17.6	0.042
Rest systolic BP, mm Hg	137.9 ± 22.7	137.4 ± 22.2	137.9 ± 22.4	0.859
Rest diastolic BP, mm Hg	79.3 ± 12.5	74.9 ± 10.9	78.6 ± 12.5	0.097
Stress systolic BP, mm Hg	132.9 ± 20.6	131.8 ± 19.8	132.8 ± 20.4	0.652
Stress diastolic BP, mm Hg	74.5 ± 12.5	74.5 ± 9.8	74.5 ± 12.2	0.763
Visual ischemic burden, %	14.7 ± 29.2 0 (0.0–12.5)	29.6 ± 28.2 25 (0–50)	17.2 ± 29.7 0 (0–25)	<0.001
MPR ischemic burden, %	6.09 ± 14.6 0 (0.0–4.9)	25.1 ± 22.0 23.4 (1.3–39.0)	8.35 ± 16.7 0 (0.0–8.5)	<0.001

Values are mean ± SD, n (%), or median (interquartile range).

BP = blood pressure; CABG = coronary artery bypass grafting; CMR = cardiac magnetic resonance; LA = left atrial; LV = left ventricular; LVEF = left ventricular ejection fraction; LVEDV = left ventricular end-diastolic volume; LVESV = left ventricular end-systolic volume; MPR = myocardial perfusion reserve; PCI = percutaneous coronary intervention; RA = right atrial; RVEDV = right ventricular end-diastolic volume; RVESV = right ventricular end-systolic volume.

The median follow-up was 460 days (IQR: 190 to 869 days). Overall, 52 patients met the primary endpoint: 39 patients underwent revascularization after 90 days (n = 23 elective PCI for stable angina, at a median 196 days [IQR: 135 to 240 days] after CMR; n = 5 unplanned PCI for unstable angina, median 220 days [IQR: 140 to 704 days] after CMR; 10 patients underwent elective CABG, median 188 days [IQR: 148 to 282 days] after CMR; and 1 patient had unplanned CABG for unstable angina, 190 days after CMR). Other events consisted of nonfatal myocardial infarction in 7 cases, median 896 days (IQR: 56 to 1,160 days) after CMR (leading to unplanned revascularization in 3 subjects), 4 cardiovascular deaths at 508 days after CMR (IQR: 186 to 856 days) and 2 aborted sudden cardiac deaths (SCDs) at 16 and 443 days after CMR.

The baseline cross-validated Cox regression model found the linear predictors to be age, sex, and LGE as follows:

∼0.025⋅age+2.993⋅sex+0.616⋅LGE

The addition of visual ischemic burden as a continuous covariate yielded a linear predictor of:

$$∼0.028\cdot \text{age}+3.108\cdot \text{sex}+0.563\cdot \text{LGE}+1.423\cdot {\text{visual}}_{\text{ischemic burden}}$$

The corresponding model for MPR ischemic burden as a continuous covariate was:

$$∼0.02\cdot \text{age}+2.722\cdot \text{sex}+0.678\cdot \text{LGE}+2.490\cdot {\text{MPR}}_{\text{ischemic burden}}$$

**Figure 1:**
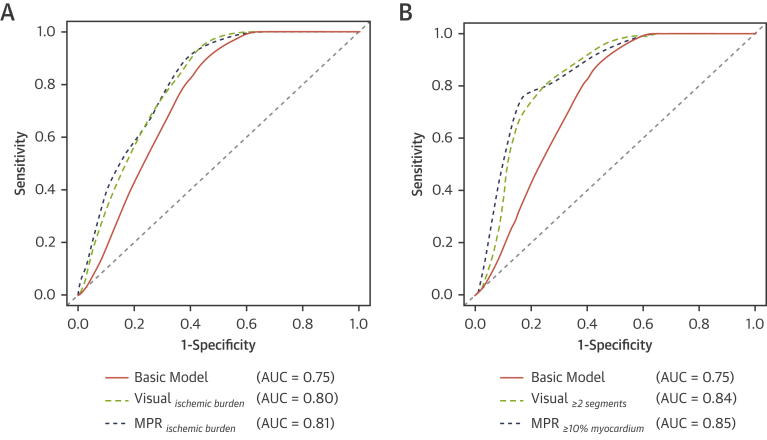
ROC Curves for Visual and MPR Ischemic Burden Receiver-operator characteristic (ROC) curves for 2-year outcome for the baseline cross-validated Cox regression model and for the extended models, including visual and ischemic burden (myocardial perfusion reserve [MPR]) as a **(A)** continuous or **(B)** dichotomized covariate.

Figure 1A illustrates the receiver-operating characteristic curves for 2-year outcome for these 3 models.

The addition to the baseline model of dichotomized visual ischemic burden using a threshold of ≥2 segments yielded a model of:

$$∼0.019\cdot \text{age}+2.949\cdot \text{sex}+0.486\cdot \text{LGE}+1.374\cdot {\text{visual}}_{\geq 2\text{ segments}}$$

The corresponding model for dichotomized MPR ischemic burden using a threshold of ≥10% ischemic myocardium was:

$$∼0.016\cdot \text{age}+2.486\cdot \text{sex }+0.497\cdot \text{LGE}+1.761\cdot {\text{MPR}}_{\geq 10\text{\%}}$$

**Figure 2:**
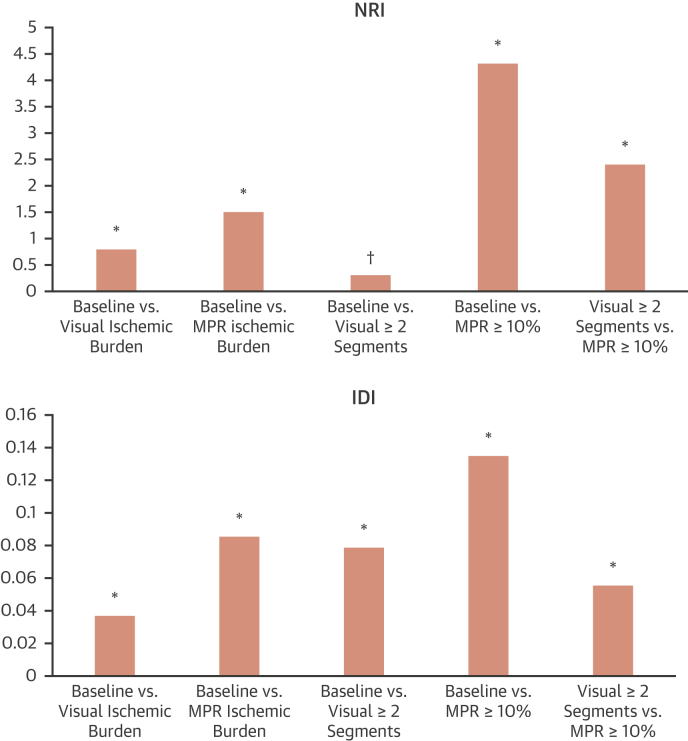
Cross-Validated Categorical NRI and IDI Reclassification for the Baseline and the Perfusion Models *p < 0.0001, †p = 0.0009. NRI = net reclassification improvement; IDI = integrated discrimination improvement; other abbreviation as in Figure 1.

**Figure 3:**
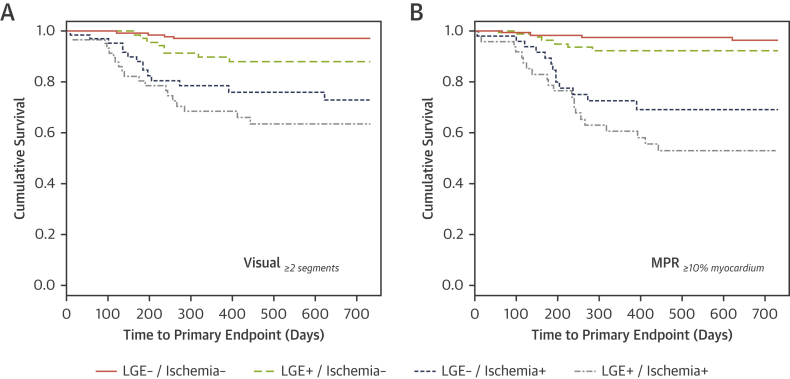
Event-Free Survival Separated According to the Presence of Significant Visual or Quantitative Ischemic Burden and LGE Kaplan-Meier curves illustrating survival in patients stratified according to late gadolinium enhancement (LGE) and **(A)** dichotomized visual and **(B)** quantitative ischemic burden. Abbreviation as in Figure 1.

Ischemia assessment by visual or quantitative analysis significantly improved predictive performance in comparison to the baseline model alone. When established clinical thresholds were used, there were further significant improvements in model performance (Figure 1B), which translated into a significant improvement in risk reclassification (Figure 2). Kaplan-Meier curves illustrating survival in patients stratified according to LGE, and dichotomized visual and quantitative perfusion findings are shown in Figure 3.

On further analysis of the concordance of results obtained from visual and quantitative analysis, the incidence of primary events was noted to be highest in the context of concordant positive tests (53.9% of the total events). The lowest event rate was observed in patients with negative results for both tests (9.6%). In cases of disagreement, we observed a higher number of patients meeting the primary endpoint when classified as positive on quantitative analysis (23.1%) compared with those who were positive only on visual assessment (13.5%).

## Discussion

The present study represents the first to address the prognostic benefit of quantitative analysis of stress perfusion CMR. A quantitative approach was previously shown to significantly improve diagnostic performance over visual assessment specifically in the setting of multivessel coronary artery disease (20). Our data demonstrated that a quantitative approach was also superior to a visual assessment in an unselected group of patients from a prognostic perspective. Moreover, our data demonstrated the incremental prognostic value of ischemic burden measurements by visual or quantitative assessment over established risk factors, including LGE, which highlighted the strength of CMR as a multiparametric technique.

In this study, both visual and quantitative analysis were performed in a high-volume tertiary center and by experts. It was a significant finding that the semi-automated quantitative analysis performed similarly, if not slightly better, than visual assessment performed by expert readers. This is of increasing relevance because recent technical advances in image reconstruction and analysis techniques are likely to permit robust full automation of quantitative analysis in the coming years 37, 38. The findings of this study have important implications for facilitating more widespread adoption of stress perfusion CMR by less experienced readers and allowing the prognostic value of perfusion quantification to be realized.

It was encouraging that there were only a small number of nonanalyzable cases (3.5%), mainly due to respiratory motion, which is known to be a limitation of the high-resolution *k-t* accelerated techniques used in this study (39). The advent of novel motion correction techniques will likely ameliorate this further (40).

We found that the consensus-based threshold of ≥10% ischemic myocardium or ≥2 abnormal segments could be validly extrapolated from nuclear medicine to CMR (16). The use of these thresholds not only improved model predictive performance, but also translated into significant reclassification of patient risk using established risk categories. This was a reassuring finding because important studies such as MR-INFORM and the ISCHEMIA (International Study of Comparative Health Effectiveness with Medical and Invasive Approaches) trial used these criteria 16, 17.

In the present study, abnormal perfusion was defined by an MPR <1.5. This threshold was previously validated against fractional flow reserve (18) and was similar to the optimal threshold found in some PET studies in patients with angina who require revascularization (41).

### Study limitations

This was a single-center study, although it took place at a tertiary hospital with a high-volume CMR service. This likely introduced a selection bias despite the enrollment of consecutive patients. However, a single-center design allowed standardization of pharmacological stress protocols and acquisition methods. A dual-bolus approach was used, justified by the need to minimize signal saturation effects in the arterial input function. Although the relative complexity of this approach makes it more difficult for less experienced centers to adopt, the emergence of dual-sequence acquisition schemes and other advances might render this less of a barrier in the future (42).

Mirroring previous clinical studies, in which visual CMR results could have influenced revascularization decisions, we excluded early revascularization events (within 90 days) from the primary composite endpoint (24). All scan reports were issued within 5 days, with a median time between scan to early revascularization of 36 days. As an additional precaution, to assess any potential bias this might have had on our results, we forced early revascularization as a covariate in our models and found that it had no impact on our findings. On this basis, we believed that the cutoff of 90 days appeared to be reasonable in our cohort for minimizing the influence of visual CMR results on revascularization decisions. Furthermore, we also proposed if there were any bias present, this would have favored a visual analysis approach because this was used for clinical decision making. We recognize that we could not fully exclude the possibility that the impact of ischemia might have been underestimated as a result of prompt revascularization.

The presence of severe ischemia, defined as vasodilator-induced systolic dysfunction by CMR, was previously shown to predict poor prognosis in a large series of patients and to identify a subgroup of patients who benefitted most from revascularization 43, 44. A comprehensive evaluation of the ischemic cascade, including induced-systolic dysfunction, was not performed in this study.

Finally, this study focused solely on perfusion CMR and used visual assessment as the clinical reference standard. CMR perfusion was not directly compared with other noninvasive modalities. PET remains the noninvasive reference method for quantitative perfusion measurements. However, it is not widely available, is costly, and uses ionizing radiation. SPECT is more widely used, but it also uses ionizing radiation and has lower spatial resolution than CMR. Visual and quantitative CMR analysis were shown to perform similarly or better than these modalities in other studies, but a direct comparison was not possible in this study 7, 19.

## Conclusions

This study supported the use of the current consensus-based prognostic ischemic burden thresholds for perfusion CMR. Quantitative perfusion analysis performed similar to, or when using a threshold-based approach, better than visual assessment. This finding represents a potentially important step forward in the goal of translating quantitative CMR perfusion analysis to the clinical setting. Our data support the need for larger, multicenter prospective randomized-controlled studies to further explore the prognostic implications of quantitative CMR perfusion analysis.

Perspectives **COMPETENCY IN MEDICAL KNOWLEDGE:** The evaluation of ischemic burden by stress perfusion CMR using either visual or quantitative assessment provides incremental prognostic value over established risk factors, including scar on late gadolinium enhancement imaging. The consensus-based threshold of ≥10% ischemic myocardium or ≥2 abnormal segments in widespread clinical use can be validly extrapolated from nuclear medicine to stress perfusion CMR. Quantitative analysis of perfusion CMR provides improved predictive power of events and reclassification of patients compared with visual analysis.

**TRANSLATIONAL OUTLOOK:** This represents an important step in supporting the translation of quantitative analysis of perfusion CMR to the clinical setting. Randomized trials are needed to validate these observational data and confirm the prognostic impact of a quantitative CMR-based strategy for guiding revascularization decisions.
